# Rapid Evidence Assessment of the Literature (REAL^©^): streamlining the systematic review process and creating utility for evidence-based health care

**DOI:** 10.1186/s13104-015-1604-z

**Published:** 2015-11-02

**Authors:** Cindy Crawford, Courtney Boyd, Shamini Jain, Raheleh Khorsan, Wayne Jonas

**Affiliations:** Samueli Institute, 1737 King Street, Suite 600, Alexandria, VA 22314 USA; Samueli Institute, 2101 East Coast Hwy., Suite 300, Corona del Mar, CA 92625 USA

**Keywords:** Rapid Evidence Assessment of the Literature (REAL), Methodology, Systematic review process, Meta-analysis, Evidence-based medicine, Scientific Evaluation and Review of Claims in Health Care (SEaRCH)

## Abstract

**Background:**

Systematic reviews (SRs) are widely recognized as the best means of synthesizing clinical research. However, traditional approaches can be costly and time-consuming and can be subject to selection and judgment bias. It can also be difficult to interpret the results of a SR in a meaningful way in order to make research recommendations, clinical or policy decisions, or practice guidelines. Samueli Institute has developed the Rapid Evidence Assessment of the Literature (REAL) SR process to address these issues. REAL provides up-to-date, rigorous, high quality SR information on health care practices, products, or programs in a streamlined, efficient and reliable manner. This process is a component of the Scientific Evaluation and Review of Claims in Health Care (SEaRCH™) program developed by Samueli Institute, which aims at answering the question of “What works?” in health care.

**Methods/design:**

The REAL process (1) tailors a standardized search strategy to a specific and relevant research question developed with various stakeholders to survey the available literature; (2) evaluates the quantity and quality of the literature using structured tools and rulebooks to ensure objectivity, reliability and reproducibility of reviewer ratings in an independent fashion and; (3) obtains formalized, balanced input from trained subject matter experts on the implications of the evidence for future research and current practice.

**Results:**

Online tools and quality assurance processes are utilized for each step of the review to ensure a rapid, rigorous, reliable, transparent and reproducible SR process.

**Conclusions:**

The REAL is a rapid SR process developed to streamline and aid in the rigorous and reliable evaluation and review of claims in health care in order to make evidence-based, informed decisions, and has been used by a variety of organizations aiming to gain insight into “what works” in health care. Using the REAL system allows for the facilitation of recommendations on appropriate next steps in policy, funding, and research and for making clinical and field decisions in a timely, transparent, and cost-effective manner.

## Background

Evidence is the basis from which we tell truth from fiction in the natural world and determine value in health care claims. Millions of articles are published in thousands of biomedical journals worldwide [1]. PubMed, a free resource developed and maintained by the US National Library of Medicine (NLM), at the National Institutes of Health (NIH), is comprised of over 20 million citations for biomedical literature from MEDLINE, life science journals, and online books [2]. With the emergence of other journal citation resources that are freely available, health care providers, consumers, researchers, and policy makers find themselves inundated with unmanageable amounts of new information from health care research. Most individuals do not have the time, skills and resources to find, appraise and interpret this evidence, nor to incorporate their findings into health care decisions in an appropriate manner. Even in special interest areas that are smaller and more narrowly focused (e.g. liver disease), it is still challenging to stay abreast with all relevant information. Consequently, despite the need for evidence to clearly inform clinical practice and policy, the best evidence is not always used due to lack of knowledge, time, skills and resources needed to quickly synthesize such information and translate that information into meaningful knowledge that can inform practice decisions.

### From clinical judgment to systematic evidence evaluation

Effective health care decisions should be evidence-based rather than rely solely on clinical judgment. Such judgments are often made under conditions of uncertainty [3], and use informal methods which can be fraught with bias and inaccuracy that produce shifting or misleading recommendations in practice. For example, as of 2012, 48 documented controlled trials and seven high quality systematic reviews (SRs) examining the effects of acupuncture on approximately 7433 total participants with substance abuse, (e.g., alcohol, cocaine, crack, nicotine dependencies and other addictions) existed in the peer-reviewed literature. Since acupuncture is widely used for substance abuse and there have been many studies done on this topic, Samueli Institute in 2012 conducted a review of SRs to summarize this evidence and concluded that, based on the current available literature, needle acupuncture was not effective in treating these conditions [4]. The implications of this review state that acupuncture is not recommended as a therapy for this condition at this time. A now classic example of the limitation of clinical judgment and the need for best evidence synthesis is in the use of hormone replacement therapy (HRT). Extensively used for years in post-menopausal women, clinicians made claims about the benefits of HRT for heart disease, sexual function, hot flashes, reduction of bone loss and prevention of cognitive decline. Subsequent randomized controlled trials (RCTs) and SRs, however, demonstrated that not only were the vast majority of these claims false, but the routine use of HRT was likely harmful [5]. Similarly, invasive laser procedures continue to be widely used for the treatment of angina from coronary artery disease (CAD) yet SRs of RCTs have shown no benefit of such procedures compared to sham controls and have reported infrequent but serious adverse events and/or interactions [6–10]. Should clinicians continue to perform these procedures? Clinical judgment is also often mis-leading or false when used by itself and the need to integrate best evidence syntheses and a method for translation of the evidence to support judgments is apparent. Without rigorous, transparent and reproducible SR processes to synthesize the best evidence, however, it is difficult to judge the efficacy, effectiveness and safety of a health care claim, identify where the gaps lie to improve the science and make appropriate decisions concerning clinical practice.

### From information to knowledge

Mastering and managing the recent explosion of medical information is a difficult task, and evidence-based problem solving skills are essential for responsible decision-making, maintaining quality health care and ensuring good outcomes. As stated, SRs form the foundation for evidence-based medicine by collating all empirical evidence that fit pre-specified eligibility criteria in order to answer a specific research question. While expert opinions and narrative reviews are popular means for organizing data, and can be informative and produced faster and more easily than SRs, they are often subjective and prone to bias. Thus, during a time characterized by large amounts of information and the critical need to make evidence-based decisions, the shift from these analyses towards SRs is not only becoming prominent, but also necessary. Indeed, high quality SRs that clearly summarize evidence have become a crucial component in helping clinicians, patients, and policymakers make accurate decisions about clinical care [3]. SR methodology holds a key position in summarizing the state of current knowledge and disseminating findings of available evidence [3, 11]. In fact, multiple groups such as the Institute of Medicine, the Agency for Healthcare Research and Quality (AHRQ), Cochrane, as well as professional associations, insurance agencies and licensing bodies that provide health care guidelines and recommendations often utilize SR methodology as a basis to offer such recommendations. Having access to and sharing high quality evidence-based SR reports within a particular subject area can help all parties be better informed about the safety, efficacy and effectiveness of treatment claims and make sound, informed decisions. They are an important step in moving from data—to information—to knowledge, provided they are conducted in a transparent, rigorous and meaningful fashion.

### Challenges with current systematic review methodology

#### Inconsistent review standards and processes

SR methodology used to assess the quality of available literature has gradually improved over the years, with several groups receiving international attention for the development of standards and advancing the science in SRs. Despite this progress, SR methodologies can still present challenges. First, many still vary considerably, and as such, outside reviewers often have difficulty replicating such methodologies. There is a need for improved standardized and reliable protocols and procedures to ensure transparency and produce meaningful information. Second, research questions and data extraction can be chosen without the input of diverse stakeholders, resulting in a narrow scope of the review, and sometimes minimal relevance or utility for making clinical decisions. Third, the subjective nature of quality assessment of research can leave SRs open to bias, resulting in unreliable results. Finally, while SRs help to provide a summary of the evidence, not all provide informative syntheses, perhaps because they lack a structured approach for obtaining expert input on the implications of the evidence for recommendations.

#### Resources

SRs can be cumbersome to execute and quite costly, requiring large amounts of personnel time and budget. Many people grossly underestimate the amount of time needed to perform a comprehensive, rigorous, and evidence-based SR, and subsequently choose to rely on less reliable methods such as expert opinions or narrative reviews. Protocol development, search strategy formation and literature searching, quality assessment and data extraction, discussion of disagreements for study inclusion, coding and quality assessments, acquisition of missing data from authors, and data analysis are all time consuming steps requiring specific skills, training and effort. A large team trained in specific roles/responsibilities at each phase of the review is needed to perform a SR most efficiently. Because lack of resources is sometimes a challenge, training, explicit processes, and the application of online systems can enhance efficiency and decrease cost. The methodology described below incorporates such methods and in turn reduces costs while enhancing the quality of the review.

### Addressing challenges of systematic review methodology

#### Samueli Institute’s Rapid Evidence Assessment of the Literature

In order to overcome these challenges and maximize efficiency in the execution and dissemination of good evidence, there is a need for more objective, high quality and up-to-date syntheses provided in a more streamlined manner regarding health care interventions. To fill this need, Samueli Institute has developed a SR process known as the Rapid Evidence Assessment of the Literature (REAL^©^). This method utilizes specific tools (e.g., automated online software) and standard procedures (e.g., rulebooks) to rigorously deliver more reliable, transparent and objective SRs in a streamlined fashion, without compromising quality and at a lower cost than other SR methods.

**Fig. 1:**
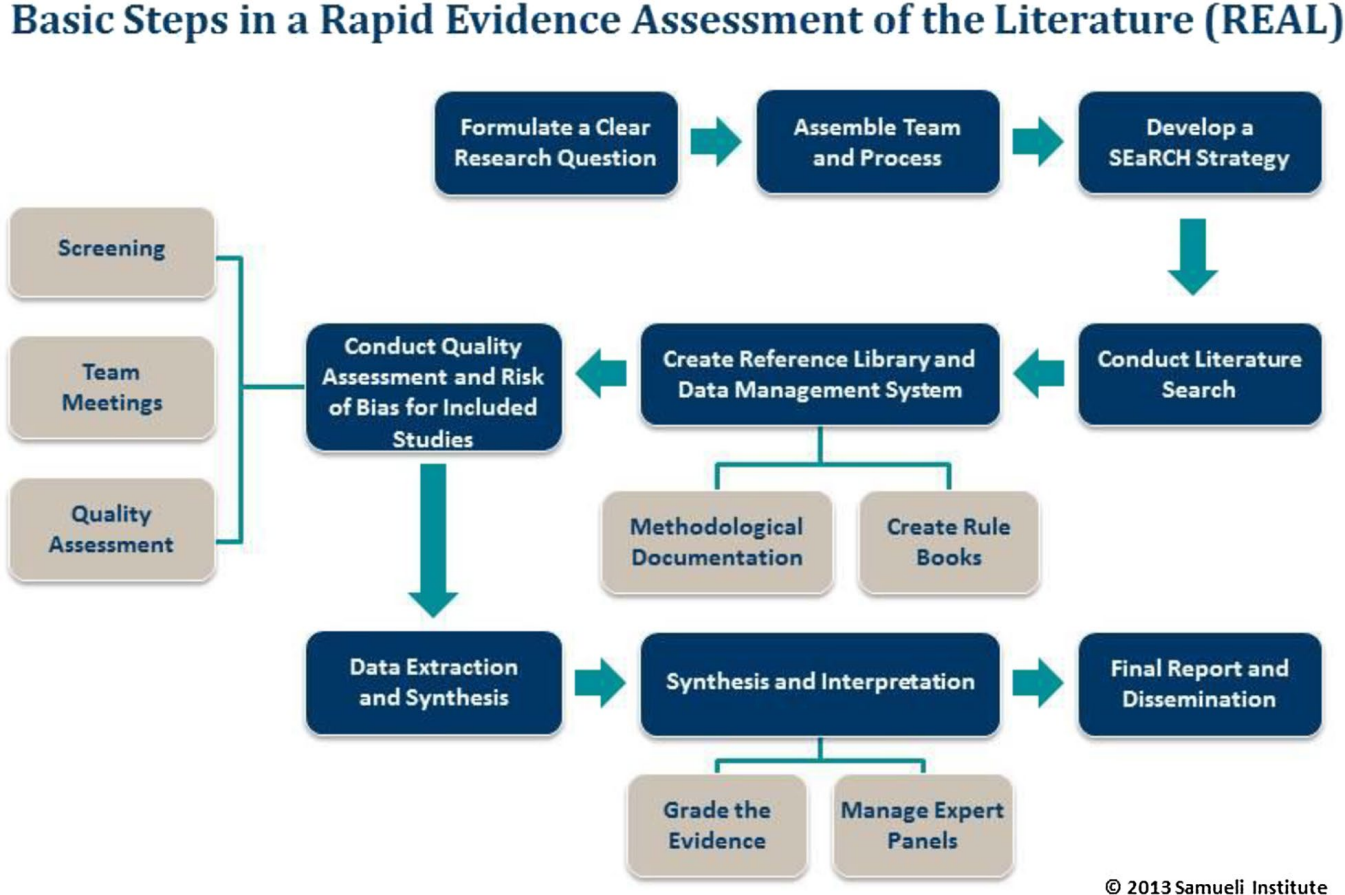
Basic steps of a Rapid Evidence Assessment of the Literature (REAL^©^)

Specifically, the REAL SR process involves (1) the rapid identification of literature relevant to a particular subject matter area (usually related to an intervention for a particular outcome); (2) the use of one or more grading systems to assess the quality and strength of evidence for the topic; (3) a summary of that evidence and; (4) subject matter experts (SMEs) input and assessments of implications for the current use of the intervention in practice. This rapid methodology requires a team-based approach to capitalize on resources and ensure maximum meaning, impact and utility; efficient and consistent review methodologies aimed at reducing time while maintaining quality; careful creation of objective protocols describing how to execute SR processes to ensure both reliability and reproducibility; as well as thoughtful synthesis and interpretation of the data to form a foundation for future work. Consequently, SRs that utilize this more streamlined process (i.e., “REALs”) are more efficient and reliable than some other traditional SR methods. Figure 1 depicts the steps involved in the REAL SR process, also detailed in the remainder of this paper. The REAL process can be used to evaluate interventions or claims in many fields including conventional medicine, complementary and alternative medicine (CAM), integrative health care (aka integrative medicine, IM), wellness, and health promotion, resilience and performance enhancement areas and more. In fact, to date, the REAL process has been applied to several topical areas [4, 12–16] with more recent published work including a Department of Defense (DoD) funded SR of reviews on acupuncture for the treatment of trauma spectrum response (TSR) components [4], self-care and integrative health care practices for stress management [15], self-care and integrative practices for the management of pain [14] and warm-up exercises for physical performance [13].

## Real methodology and design

### Following a team-based approach to capitalize on resources

Efficiency is of great importance when stakeholders need immediate, evidence-based answers for “what works”. Many review teams are small in size and reviews can take years to complete. Conversely, to maximize efficiency, Samueli Institute REALs are executed by several well-trained team members, each with specific roles and responsibilities, and often take approximately 3–6 months, from question development to manuscript delivery.

Specifically, a REAL Review Team includes: (1) a *Principal Investigator* to oversee the entire project; (2) a *Review Manager* with SR methodology expertise to guide the review process from start to finish; (3) a *Search Expert* to assist with literature search strategy development and execution; (4) at least two trained *Reviewers* to screen, extract data and review the quality of the literature; (5) a *Reference Manager/Research Assistant* to provide administrative and project support; (6) a *Statistician* to provide guidance regarding the interpretation of complex results or meta-analyses; and (7) at least two *SMEs* with diverse perspectives related to the review topic to provide guidance and synthesize the overall literature pool. It is important to note, that while Samueli Institute has designed the REAL process to be executed by individuals within these roles/responsibilities, some organizations and entities may be more limited in terms of available personnel. As such, it is reasonable for individuals to be trained to take on multiple roles, although doing so may delay the review process. The division of labor allows for more efficient, accurate and reliable execution of the review steps and reduction of time needed by any one individual. Further, it allows for better compliance with the Institute of Medicine (IOM) recommendations for managing bias and conflicts of interest (COI) when producing reviews and recommendations [3]. The REAL process follows these IOM recommendations and follows strict criteria at each review step to guard against bias and excludes team members with COIs from portions of the review where objectivity or balance may be compromised.

### Involving stakeholders to ensure maximum relevance and translatability

One of the most frequent complaints by clinicians and patients about systematic reviews is that their conclusions have little relevance to daily clinical decisions and so are not of much use. The REAL has built in a process to obtain continuous input from any stakeholder involved in these decisions. In addition to the Review Team, REALs also include a *Steering Committee* comprised of 4–6 diverse stakeholders (e.g., clinicians, researchers, policy makers, patients and various other relevant stakeholders) chosen by the client and Principal Investigator, who provide guidance throughout the review process. This ensures that the review’s focus stays relevant to the end-user of the SR results and allows for translation to practice to occur more effectively. The Steering Committee seeks to address the “so what” question that so often occurs after a standard SR in which simply “more and better research is needed.” Though integral to the review, the Steering Committee is not involved in the review’s technical steps. This guards against bias during the independent evidence assessment process. Once the Steering Committee and the SMEs review and approve the team’s plans and progress at each review phase the Review Team is then solely focused on conducting the review and analyses in an independent and objective fashion.

Once assembled, it is imperative that both the Review Team and Steering Committee work together to formulate the review’s research question, scope, definitions, and eligibility criteria using the PICO(S) process (i.e., Population, Intervention, Control or Comparison, Outcomes and Study Design) [2], as well as identify relevant data extraction points for synthesizing the literature. Assembling various stakeholders to pre-define the review’s research question and eligibility criteria sets the tone for the review, ensuring that different perspectives are represented in the review and requiring that all subsequent steps and processes are conducted with this information in mind. This is a critical part of the REAL and ensures the results will have enough meaning and utility for stakeholders. Although involving a large group of voices at the outset to deliberate and agree upon all elements of the SR may seem counterintuitive to increasing efficiency, outlining a clear methodological process up front is imperative to streamlining the remaining systematic processes and so saves time overall. In addition this reduces the chance that the team will have to redefine their research question or processes once the review is underway. Revisions done while the quality assessment of the literature is underway not only costs time and resources, it opens the process to bias. These are reduced in the REAL process.

### Enhancing the efficiency and consistency of review methodologies

#### Utilizing specific search protocols to reduce quantity and improve quality

The REAL process requires search expertise to build robust literature search strategies as well as iterative input from both the SMEs and Steering Committee members for guidance. REALs do not “exhaustively” search the literature by including grey and non-English language literature, unless essential to the specific research question (e.g., searching Chinese herbal therapy). Instead, they usually include only peer-reviewed literature published in the English language. While the traditional SR considers the inclusion of only English-language studies as a limitation, doing so rarely compromises the outcome or implication for the majority of interventions and claims [17]. There has been debate, moreover, around the importance of including grey (unpublished) literature. While including such literature can reduce publication bias, it can also result in the overestimation of an intervention’s effects, since unpublished studies are usually more difficult to find, smaller and of lower quality compared to those published in the English language literature [18, 19]. Therefore, despite the inherent differences in methods as well as time and cost associated with these processes, the conclusions of a REAL and a SR are usually comparable, and result in the same “bottom line” conclusions about the evidence [20]. In fact, the synthesis involved in a REAL is often more informative and rigorous than some SR efforts due to the additional assessment systems employed in a REAL compared to standard SRs [4, 21] (see *Adapting and Developing of Quality Assessment Tools*).

#### Automating the review to enhance the review process

REALs are more efficient due to not only their focus on English and peer-reviewed literature, but also their use of readily available software systems to automate the review process. These systems have been customized for use with a REAL and streamline many of the review steps including automated article processing and management, eliminating the need for data transcription, automated reliability estimation, real time error and quality checking, and reduction of post-review data collation. Using a specific review system and rulebooks allows researchers to deliver results faster, with improved accuracy and reliability, and provides a complete audit trail of all changes to ensure transparency. Such systems can also be accessed remotely and include messaging features that allow the review team to interact virtually, thereby considerably decreasing costs associated with travel, materials, supplies, and meeting facilities.

### Ensuring objectivity to reduce bias

#### Adapting and developing of quality assessment tools

Most groups using SRs to develop recommendations and guidelines rely on subject matter experts (SMEs) to evaluate the quality of the research. However, SMEs almost always have a particular point of view (bias) and also are rarely trained in the proper use of quality assessment tools. REALs avoid the use of SMEs in applying quality assessment tools and instead rely on trained review teams. This way, higher standards for accuracy and reliability are obtained. There are many well-accepted quality assessment rating systems available to researchers for evaluating quality and risk of bias. These tools typically focus on internal validity, or whether the results are due to attributional bias issues. These tools are usually quite subjective and variable in how quality criteria are interpreted. Samueli Institute has adapted some of these rating systems to improve their use and objectivity. In addition, we have also developed, validated and incorporated an External Validity Assessment Tool (EVAT^©^) [16] into the REAL process to assess the “real-world” relevance of the research questions being asked. While many SRs typically only evaluate internal validity, REAL uses quality assessment tools to evaluate not only internal validity but external and model validity as well. Thus, all REALs deliver a database of ratings for gaging the attributional (internal validity), generalizability (external validity) and relevance (model validly) of every study. This database has multiple uses for clients even after the specific REAL is completed.

#### Detailing and applying quality criteria

Due to the inherently subjective nature of interpreting research results, Samueli Institute has created rulebooks to ensure that review teams are: (1) objectively evaluating and “scoring” each included article for quality; and, (2) consistently extracting data in a specific, consistent format, thereby reducing time needed for post-review data cleaning. Reviewers utilize these rulebooks and so provide transparent data extraction as well as a consistent and sufficient inter-rater reliability Cohen’s Kappa (i.e., 90 %), indicating a low level of conflict and high level of agreement between reviewers. These rulebooks are essential for managing and minimizing bias and ensuring the quality of any review. For example, should someone question the basis for any results in a SR, the team can refer to the rulebooks to explain and demonstrate specifically why and how particular articles were scored.

#### Maintaining transparent reporting

Just as the criteria and parameters whereby reviewers conduct the review are explicitly detailed in rulebooks, all decisions, processes and outcomes relating to each step of the review are maintained in a Review Documentation Checklist throughout the review process. Because this Checklist was developed to adhere to the Preferred Reporting Items for Systematic Reviews and Meta-Analyses (PRISMA) Guidelines [22], it not only aids with transparent reporting of results and replication of methods, but can also be used as a guide for how the results can be synthesized into a report and disseminated through peer-reviewed journal publications or other venues. Using this checklist for a manuscript outline also streamlines manuscript preparation as authors have all methodological processes and decisions housed in one place, rather than having to dig through files to find the details from various phases of the review [22].

#### Synthesizing and interpreting the data to find meaning

The REAL process is designed to provide a basis for SMEs to identify current implications for research and practice based on the evidence as a whole. In fact, once all individual studies included in the review have been evaluated, SMEs assess the overall literature pool according to the researched outcomes relevant to the research question in order to: (1) determine the quality of the research as a whole; (2) identify gaps in the literature; (3) assess the effectiveness of the intervention or claim as well as the confidence in that effectiveness estimate; and (4) judge the appropriateness for clinical use of the intervention. This is done in the following way. A roundtable is convened with the review team, Steering Committee and SMEs to evaluate the review’s results, the overall literature pool analyses, identified gaps, as well as outline next steps for the particular field of research. Several tools are used to organize the goals and discussion at this roundtable. A synthesis report is produced from this roundtable that is reviewed and modified by the REAL team based on feedback from all participants. These syntheses form a foundation for researchers, clinicians and patients to be better informed about the current state-of-the-science for any intervention, and determine next steps needed in the field of research and practice for use and impact. The Grading of Recommendations, Assessment, Development and Evaluation (GRADE) working group has developed methods for going about synthesizing the literature as a whole that should be used standardly across all systematic reviews [23].

### Laying the foundation for evidence based decisions

REALs are constructed in a way that lays a foundation for future stakeholders to use quality evidence for decision making in multiple areas—research, practice, personal and policy areas. These foundational elements include the evaluated dataset (which can be further updated and added to), effect size estimates, meta-analyses (when possible), and other elements that go into the report such as the quality tools previously described and synthesis and interpretation assessments.

#### Conducting meta-analyses

Meta-analyses combine the actual quantitative results (e.g., collect and pool effect sizes) of separate studies included in a review, use statistical techniques to determine the overall effect size and confidence in the effect of the intervention, and employ analytic techniques to quantify possible publication bias. They are often costly and time-consuming, and only appropriate when the existing literature suggests that there are sufficient studies with enough homogeneity in outcomes. REALs are designed to form the foundation for subsequent meta-analyses to be conducted, if appropriate. REALs can therefore be utilized as an effective tool for rapidly determining the current state of the literature, and what gaps should be addressed to conduct an effective meta-analysis.

#### Bridging the gap between evidence and knowledge

There is a considerable barrier to rapidly translating evidence into decision making for clinicians, patients, researchers and policy makers. Although authors of SRs disseminate results through various routes of publication, results often do not reach *all* parties in ways that allow them to make medical decisions and so do not maximize impact of reviews. The REAL process is one of three components of Samueli Institute’s Scientific Evaluation and Review of Claims in Health Care (SEaRCH) Program and is a key step to forming a foundation upon which the other two SEaRCH components can be used for determining the clinical impact and relevance of evidence. SEaRCH is comprised of the REAL, Claim Assessment Profile (CAP) and Expert Panel Processes (e.g., Clinical, Research and/or Policy Expert Panel) processes as described in this journal issue [24, 25]. Together these three segments of SEaRCH can be integrated with each other in order to answer the question of “what works” in health care by providing: (1) a clear description of the intervention and claim being evaluated and its feasibility to engage in future research through the CAP; (2) a rigorous summary of current evidence for the claim gathered through the REAL process and shared with the other components of SEaRCH; (3) a balanced, expert assessment of the appropriateness of use of the intervention with the Clinical EP; evidence-based policy judgments needed to direct implementation of a practice claim with the Policy EP; the value of the research for patient-centered care with the Patient EP; and (4) next research steps needed to move the evidence base about the claim forward with the Research EP. The methods used for the CAP and the EPs process are described in subsequent articles in this set.

Similar to the REAL, expert panels and the CAP employ specific processes and safeguards to reduce variability and bias and promote collaboration and efficient delivery of meaningful results. The CAP can be conducted prior to the REAL to inform the REAL toward specific definitions about a particular claim, or can be conducted in tandem with the REAL for informing the expert panel process. While expert panels can be organized once the REAL process is completed it is important that the review and expert panel processes both remain independent of each other to manage bias and maintain a focus on clinical and patient relevance. To do this properly, SME input and the REAL process need to be carefully managed even as they are linked to the expert panel process. The SEaRCH program is designed to allow for complete interaction between the SR and expert panel process in a manner that remains both impartial and informative in the interaction between SMEs and the trained reviewers [3]. This process creates distinct, independent teams who not only engage in the literature review and expert panel processes, but also “cross-talk” (under the supervision of a SEaRCH Program Manager and the Steering Committee chair) to ensure that both relevant research questions are being addressed and the rigor of the research is maintained. Specifically, when an expert panel is solicited, the Expert Panel Manager [26] and REAL Review Manager collaborate to ensure that the panel’s topic of interest is being sufficiently addressed by the REAL. Panelists, based on their expertise, can expand upon the gaps or clinical issues brought forth through the REAL. REALs can assist expert panels to determining appropriateness, clinical guidelines, implementation policies and patient-centeredness of the evidence or for establishing research agendas. Recommendations that emerge through the SEaRCH process can then be shared with stakeholders for maximum impact.

### Summary

The REAL is a process that streamlines and organizes many elements of systematic reviews in order to insert high quality, rigorous evidence in a more rapid, objective, relevant and cost-efficient manner into decision making processes. Specifically, the REAL (1) follows a team-based approach; (2) utilizes specific search strategies; (3) automates review processes to ensure efficient use of time and skill; (4) involves key stakeholders to guarantee the right questions are being asked and addressed; (5) outlines and adheres to a transparent protocol to ensure objectivity and the management of bias; and (6) forms a foundation for subsequent analyses and expert panels to guide gaps and relevance, particularly when tied into other elements of the SEaRCH process. These features not only increase efficiency, but also assure adherence to reliable and reproducible protocols that provide a more consistent, transparent SR process for evidence-based medicine and decision making by the multiple stakeholders in health care.

By providing background and information on the existing literature, research gaps, and the weaknesses and strengths of current evidence, systematic reviews utilizing the REAL process provide a solid and consistent foundation for making clinical, patient and policy decisions. The, objectivity and efficiency of the REAL process make it a valuable for a variety of organizations and entities that need good evidence for decisions about products, practices or programs currently in use or those being explored for potential use. Decision makers as diverse as a health insurance or regulatory company/agency wanting to know what the evidence is for an intervention in order to decide whether or not it should be covered, or a clinical practice wanting to know if implementing a certain practice would benefit their patients are examples of decisions that can be aided by a REAL.

### Training and support for conducting REALs

Samueli Institute has shared their REAL methodology with others in the SR field and continues to extend outreach and support to those interested in using this approach for evidence assessment. The Institute has developed a workshop that teaches participants how to conduct SRs in the step-wise fashion used by the REAL. This workshop is currently offered 2–3 times a year and provides participants with a comprehensive workbook covering theoretical material (i.e., the role and purpose of different types of SRs, their place in delivering evidence-based medicine, role of bias, etc.), practical instructions and guidelines on how to conduct SRs using the REAL process, and allows participants to receive individual coaching on review projects they are developing or conducting. The course and assistance is also offered through an online, self-paced platform (Black Board) complemented by didactics, mentorship, and in person workshops. Samueli Institute also collaborates with other organizations wishing to evaluate a topic using the REAL methodology, and offers guidance and mentorship throughout the review process. These workshops have been done for government and private groups and can be customized for use by any organization interested in applying evidence to health care decision making.

## Discussion

There is a need for reliable, rapid, and transparent evidence to guide effective health care decision-making. The REAL approach was developed to ensure high quality SRs are conducted in a rapid, streamlined, transparent and valid fashion. It has been shown to: (1) reduce the cost of generating reviews for those making informed decisions regarding health care; and, (2) inform the public in a time sensitive, cost-effective and objective manner about the state of the evidence for any health care area.

Detailing the challenges of current SR methodology and the ways in which this rapid SR process addresses those challenges highlights the need for investigators to ensure that reviews are objective, transparent, scientifically valid, and follow a common language and structure for characterizing the strength of evidence across reviews. Adapting an approach like the REAL into current SR processes will not only decrease the variability and improve the quality of SRs, but also allow health care decision makers, including clinicians, patients and policy makers to play a crucial role in developing relevant research questions and for making sound, evidence-based decisions in all of heath care.

For those interested in utilizing the REAL approach and learning more about conducting SRs, training workshops and collaboration opportunities, please visit the Samueli Institute website [27].

## Abbreviations

AHRQ: Agency for Healthcare Research and Quality
CAD: coronary artery disease
CAP^©^: Claims Assessment Process
COI: conflict of interest
DoD: Department of Defense
EP: expert panel
EVAT^©^: external validity assessment tool
HRT: hormone replacement therapy
IOM: Institute of Medicine
NIH: National Institutes of Health
NLM: US National Library of Medicine
PICO(S): population, intervention, control or comparison, outcomes, study design
PRISMA: preferred reporting items for systematic reviews and meta-analyses
RCT: randomized controlled trials
REAL^©^: Rapid Evidence Assessment of the Literature
SEaRCH™: Scientific Evaluation and Review of Claims in Health Care
SME: subject matter expert
SR: systematic review
TSR: trauma spectrum response
